# Water-assisted and controllable synthesis of core/shell/shell structured carbon-based nanohybrids, and their magnetic and microwave absorption properties

**DOI:** 10.1038/s41598-017-10352-8

**Published:** 2017-08-29

**Authors:** Xiaosi Qi, Erqi Yang, Hongbo Cai, Ren Xie, Zhongchen Bai, Yang Jiang, Shuijie Qin, Wei Zhong, Youwei Du

**Affiliations:** 1 College of Physics, Guizhou University, Guiyang, 550025 People’s Republic of China; 2 Guizhou Province Key Laboratory for Photoelectrics Technology and Application, Guizhou University, Guiyang City, 550025 People’s Republic of China; 3 Nanjing National Laboratory of Microstructures and Jiangsu Provincial Laboratory for NanoTechnology, Nanjing University, Nanjing, 210093 People’s Republic of China

## Abstract

By controlling the pyrolysis temperature, core/shell/shell structured Fe/Fe_5_C_2_/carbon nanotube bundles (Fe/Fe_5_C_2_/CNTBs), Fe/Fe_3_C/helical carbon nanotubes (Fe/Fe_3_C/HCNTs) and Fe/Fe_3_C/chain-like carbon nanospheres (Fe/Fe_3_C/CCNSs) with high encapsulation efficiency could be selectively synthesized in large-scale by water-assisted chemical vapor deposition method. Water vapor was proved to play an important role in the growth process. Because of α-Fe nanoparticles tightly wrapped by two layers, the obtained core/shell/shell structured nanohybrids showed high stabilities and good magnetic properties. The minimum reflection loss values of the as-prepared nanohybrids reached approximately −15.0, −46.3 and −37.1 dB, respectively. The excellent microwave absorption properties of the as-prepared core/shell/shell structured nanohybrids were considered to the quarter-wavelength matching model. Moreover, the possible enhanced microwave absorption mechanism of the as-prepared Fe/Fe_3_C/HCNTs and Fe/Fe_3_C/CCNSs were discussed in details. Therefore, we proposed a simple, inexpensive and environment-benign strategy for the synthesis of core/shell/shell structured carbon-based nanohybrids, exhibiting a promising prospect as high performance microwave absorbing materials.

## Introduction

With the explosive development of information technology and rapidly expanding use of communication devices, serious electromagnetic (EM) interference pollution has become a great concern, which can cause great disturbances on the medical, industrial, commercial, military equipment and is also potentially harmful to biological systems^1–4^. Therefore, the effective microwave absorbing materials (MAMs) have attracted a great deal of attention in order to attenuate those unwanted EM energies, which is an important issue to be considered for both civil and military purposes^5–8^. Over the past decade, a variety of materials used as microwave absorbers have been extensively studied with an increasing demand for innovative EM interference shielding^9–14^. Recently, considerable attention has been paid to the development of high-efficiency MAMs with light weight, thin thickness, strong absorption characteristics and excellent antioxidant ability^15^.

In particular, carbon materials, such as carbon black, graphite flakes, carbon nanotubes (CNTs) and graphene (G), have been explored as promising MAMs, especially in the case of lightweight and harsh environment^16–19^. However, the complex permittivity $({\varepsilon }_{r}=\varepsilon ^{\prime} -j\varepsilon ^{\prime\prime} )$ and complex permeability $({\mu }_{r}=\mu ^{\prime} -j\mu ^{\prime\prime} )$ of pure carbon materials are out of balance^20, 21^. The serious mismatch in the values of ${\varepsilon }_{r}$ and ${\mu }_{r}$ will induce that most of the microwave radiation is reflected, rather than absorbed. Based on the impedance matching strategy, one of the effective ways to solve the problem is to incorporate with magnetic nanoparticles. Among these hybrids, core/shell structured magnetic nanoparticles/carbon-based nanohybrids have been proved to exhibit strong EM wave absorption properties because of their interfacial and synergistic effects^22–26^. For example, Wadhawan *et al*. reported that the greater microwave absorption properties of the single-walled CNTs with impurities of magnetic Fe nanoparticles were affected by their cooperative effect^27^. Kim and Che *et al*. synthesized core/shell structured Fe/CNTs, and proved their excellent microwave absorption capabilities, respectively^5, 28^. Zhang *et al*. reported that the Ni/C composites exhibited improved microwave absorption properties, which was attributed to the good match between the dielectric loss and magnetic loss^29^. Qu *et al*. found that the enhanced EM wave absorption ability of Fe_3_O_4_-Fe/G composite was ascribed to the well-matched characteristic impedance^30^. So far, various methods and schemes have been proposed to produce core/shell structured magnetic carbon-based nanohybrids, such as chemical vapor deposition (CVD), arc discharge, pulsed laser irradiation of solution, hydrothermal method, and so on^31–35^. Nevertheless, most of these routes available now still suffer from complicated and expensive processes, low capsulation efficiency and uncontrollable synthesis. Therefore, it is desirable to develop a simple, inexpensive and environment-benign strategy for the synthesis of core/shell structured carbon-based nanohybrids with high capsulation efficiency.

Based on the previously reported results^36–40^, in this article, we report the selective synthesis of core/shell/shell structured Fe/Fe_5_C_2_/carbon nanotube bundles (Fe/Fe_5_C_2_/CNTBs), Fe/Fe_3_C/helical carbon nanotubes (Fe/Fe_3_C/HCNTs) and Fe/Fe_3_C/chain-like carbon nanospheres (Fe/Fe_3_C/CCNSs) over Fe nanoparticles by water-assisted CVD method. The results indicate that the introduction of water vapor has a great impact on the yield and morphology of the obtained samples. Due to α-Fe nanoparticles tightly wrapped by two layers and their synergetic effect, the as-prepared Fe/Fe_5_C_2_/CNTBs, Fe/Fe_3_C/HCNTs and Fe/Fe_3_C/CCNS show high stabilities, good magnetic properties, and excellent microwave absorption performances.

## Results

**Figure 1:**
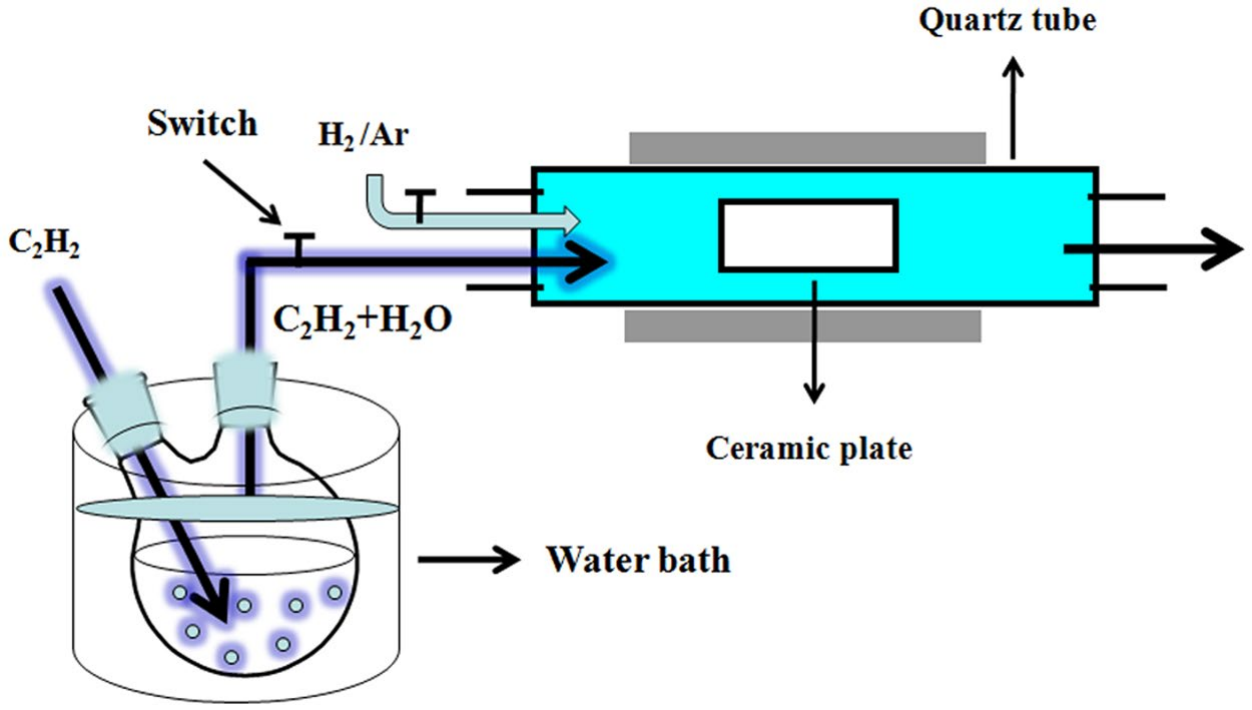
Schematic for the synthesis of core/shell/shell structured nanohybrids.

**Figure 2:**
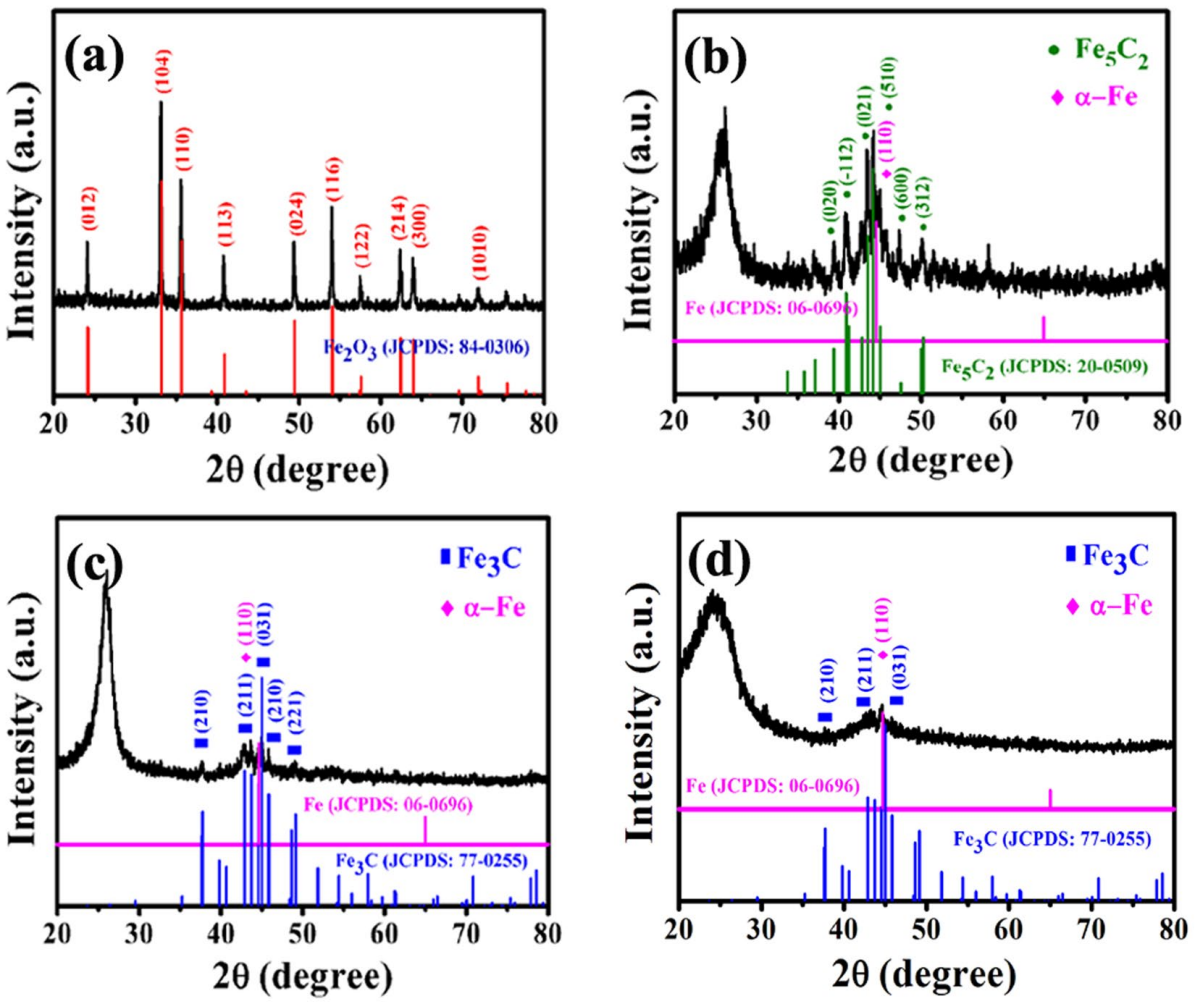
XRD patterns of (**a**) catalyst precursor, (**b**) C-400, (**c**) C-450 and (**d**) C-600.

Figure 1 presents the schematic for the synthesis of core/shell/shell structured nanohybrids. After cooling to room temperature (RT) in Ar, averagely 0.21, 1.01 and 0.86 g of the black sample could be collected in ceramic plate. For easy description, the samples generated at 400, 450 and 600 °C are denoted hereinafter as C-400, C-450 and C-600. Figure 2 shows XRD patterns of the as-synthesized catalyst precursor and samples. As shown in Fig. 2a, the diffraction peaks at 24.1, 33.2, 35.6, 40.9, 49.5, 54.1, 57.5, 62.4, 64.0 and 72.0° can be assigned to (012), (104), (110), (113), (024), (116), (122), (214), (300), and (1010) crystal planes of Fe_2_O_3_ (JCPDS No.84-0306). In the absence of signals corresponding to other phases, the result indicates that the catalyst precursor is single-phase α-Fe_2_O_3_. As indicated by the symbols in Fig. 2b, all the marked diffraction peaks of C-400 correspond to cubic phase of Fe_5_C_2_ (JCPDS No.20-0509) and cubic phase of α-Fe (JCPDS No.06-0696), respectively. And the diffraction peak at ca. 25.8° corresponding to an interlayer spacing of 0.34 nm, which is attributed to the graphite-like carbon. The XRD patterns of C-450 and C-600 (as shown in Fig. 2c and d) indicate that all the labeled peaks can be indexed to the orthorhombic phase of Fe_3_C (JCPDS No.77-0255) and cubic phase of α-Fe (JCPDS No.06-0696), respectively. And the diffraction peak of C-450 and C-600 located at 20–30° is assigned to the encapsulating carbon shell. Based on the obtained XRD results, one can find that C-400 shows higher XRD signals of Fe_5_C_2_ and α-Fe than those of C-450 and C-600, which may be related to the higher content of Fe_3_C and α-Fe in C-400 or/and the thinner encapsulation thickness^41, 42^. In the study, as revealed in experimental section, smaller quantity of C-400 could be collected than that of C-450 and C-600 with an equal weight of α-Fe_2_O_3_ (0.1 g), implying that the highest Fe content of C-400 among the obtained samples. In order to confirm the yields (defined as weight ratio of the collected sample to catalyst) of the obtained samples, each experiment was repeated three times. As shown in Table 1, it can be seen that the designed experiments show an excellent reproducibility and the experimental results are well consistent with the XRD characterizations. Compared to the previously reported Fe/HCNTs^43^, much higher yield of sample could be obtained by our proposed route.

**Table 1:** Weight of the collected samples at selected temperature in three runs.

Pyrolysis temperature (°C)	400	450	600
Sample	C-400	C-450	C-600
Weight of collected sample in each runs (g)	0.213	1.009	0.860
	0.208	1.013	0.864
	0.212	1.014	0.859

**Figure 3:**
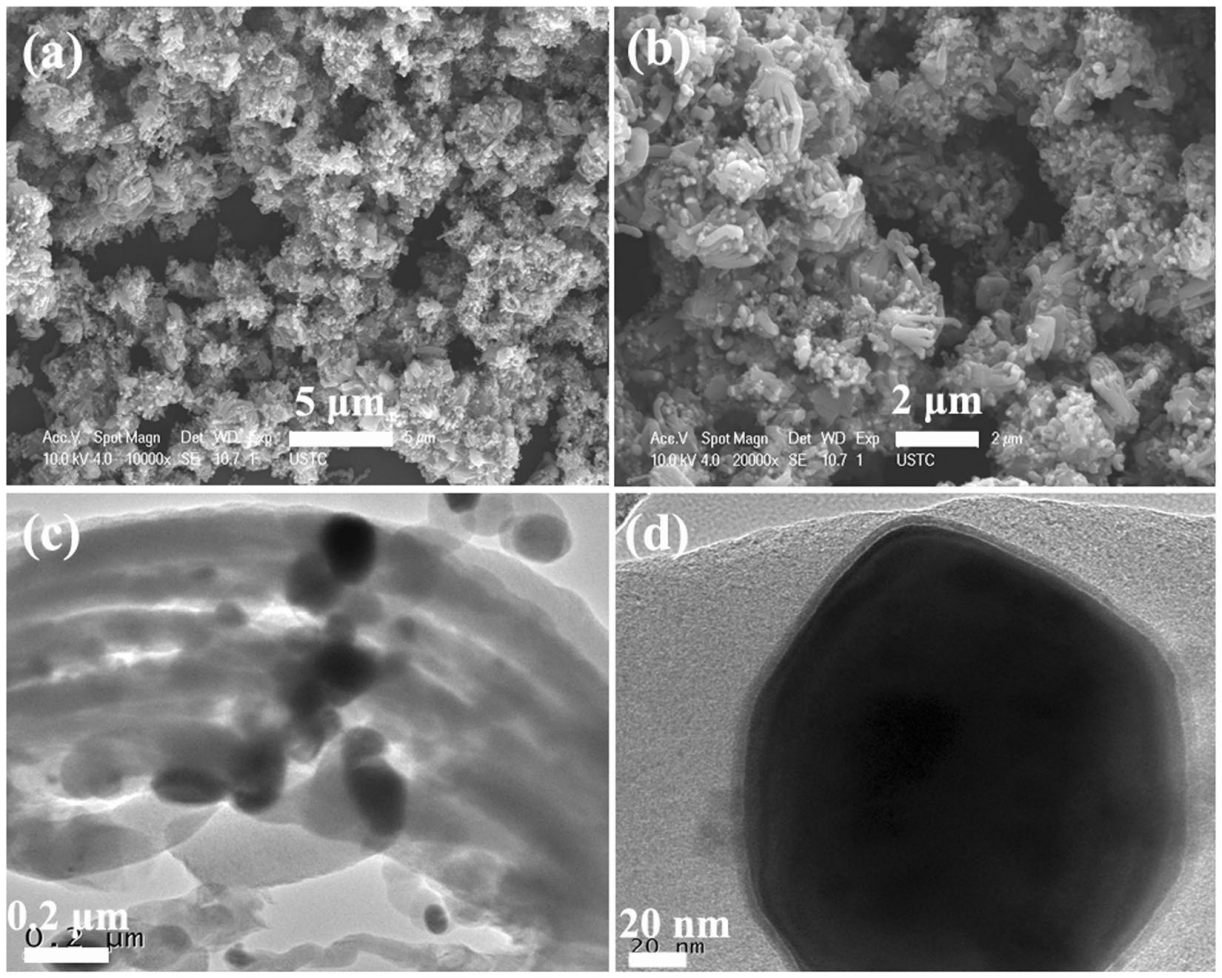
(**a**,**b**) FE-SEM, and (**c**,**d**) TEM images of C-400.

Figure 3 presents the microstructure of the as-prepared C-400. The FE-SEM observation (as shown in Fig. 3a and b) reveals that the bright nanodots, which should correspond to the catalyst nanoparticles, are well distributed throughout the obtained carbon nanotube bundles (CNTBs). The TEM investigation (as shown in Fig. 3c) indicates that the catalyst nanoparticles are tightly encapsulated into the tubes of CNTBs. Figure 3d gives a closer TEM image, which displaying evidently that the obtained C-400 exhibits three-layer structure. Based on the obtained results of XRD and microstructure, we can conclude that the as-prepared C-400 is high selectivity of core/shell/shell structured Fe/Fe_5_C_2_/CNTBs nanohybrid. In order to confirm this result, as shown in Figure S1 (in Supporting Information), the energy dispersive X-ray spectroscopy (EDS) result of the selected area indicates that the elements of C, Fe, O, Pt and Cu can be detected over the obtained C-400. In this study, combined with the sample preparation process before FE-SEM characterization, we think that the C and Fe signal originates from the as-prepared sample, O should be ascribed to the adsorption of H_2_O on the obtained sample, Cu signal comes from the copper grid and the spray Pt before the sample characterization induces the appearance of Pt signal. Moreover, The FE-SEM and TEM investigations show that the statistical encapsulation efficiency is ca. 96%. Compared with the schemes reported in the literatures^44–47^, the method adopted in this study is simple, inexpensive and high encapsulation efficiency.

**Figure 4:**
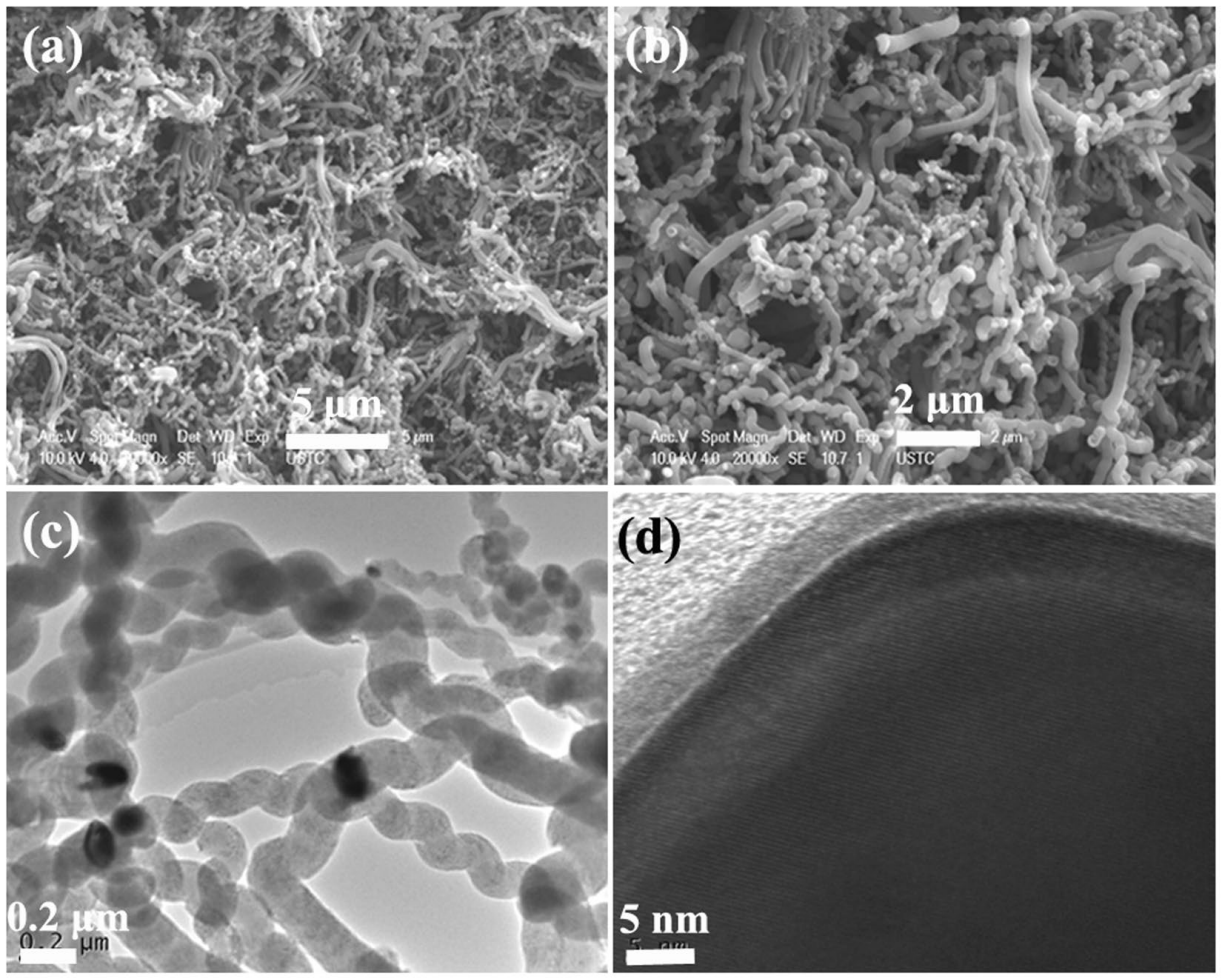
(**a**,**b**) FE-SEM, and (**c**,**d**) TEM images of C-450.

The FE-SEM and TEM images of the obtained C-450 are shown in Fig. 4. As displayed in Fig. 4a and b, large scale of helical carbon nanotubes (HCNTs) can be seen evidently and the catalyst nanoparticles (the bright nanodots) are well distributed throughout the obtained sample. Similar to that of C-400, the TEM observation of C-450 (as shown in Fig. 4c and d) indicates that the catalyst nanoparticles are tightly enwrapped by HCNTs and its characteristic of three-layer structure can also be seen clearly. Combined with the results of XRD, we can conclude that the as-prepared C-450 is high selectivity of core/shell/shell structured Fe/Fe_3_C/HCNTs nanohybrid. Compared to the previously reported results^44, 48–50^, the as-prepared C-450 exhibits an evident structure of core/shell/shell and the obtained yield is high. Moreover, The FE-SEM and TEM investigations show that the morphology of CNTBs can be observed occasionally and the statistical encapsulation efficiency is ca. 95%.

**Figure 5:**
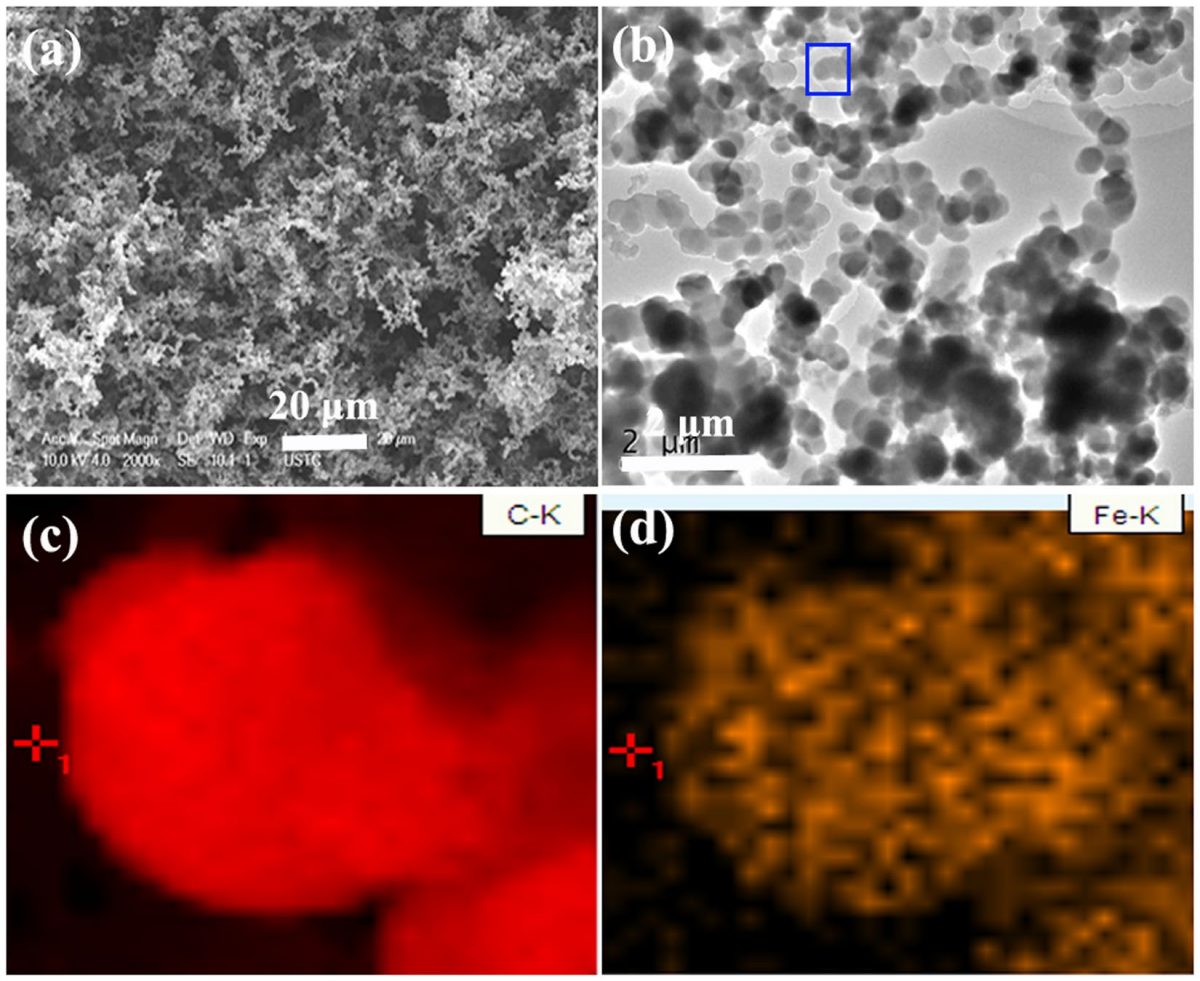
Microstructures of C-600: (**a**) FE-SEM image, (**b**) TEM image (**c**,**d**) EDS elemental mapping of C and Fe from the area as indicated by the green square in (**b**).

The microstructure of the as-synthesized C-600 is shown in Fig. 5. As shown in Fig. 5a, the FE-SEM observation reveals that the obtained C-600 is spheroidal carbon particles, and the content of such structure is up to 99%. It is apparent that these spheres have the uniform size and their average diameter is ca. 400 nm. According to the classification given by Serp *et al*.^51^, the obtained carbon material in the case can be called carbon nanospheres (CNSs). A closer TEM observation (as shown in Fig. 5b) indicates that the obtained carbon nanosphere connects with each other to form chain-like carbon nanospheres (CCNSs) and the catalyst nanoparticles cannot observed obviously. In order to confirm the existence of the catalyst nanoparticles, the selected elemental mapping are conducted. As shown in Fig. 5c and d, the results of element mapping reveal that the as-prepared CNSs are composed of C and Fe. Considering the XRD and TEM results, we think that the as-prepared C-600 should be core/shell/shell structured Fe/Fe_3_C/CCNSs nanohybrid. In order to confirm the composition and chemical state of iron, XPS measurement was performed. As shown in Figure S2a, the typical peaks at ca. 284.4 and 285.8 eV can be observed clearly, which can be attributed to the carbon layers in the obtained sample^52^. Similar to that of FeC_x_/carbon composites reported elsewhere^52–54^, the appearance of C-Fe bonding (283.7 eV) confirms the formation of Fe_3_C in the obtained C-600. As shown in Figure S2b, the Fe 2p1/2 and Fe 2p3/2 peaks centered at 709.9 and 723.2 eV can be assigned to Fe^2+^ ^52–54^, which further indicates that the existence of Fe_3_C in the obtained C-600. In addition, compared to the CNSs reported before^55–57^, the route proposed by us is a low temperature and feasible scale-up for preparing CNSs. In general, as shown in Table 2, it should be noted that the pyrolysis temperature has a great impact on yield and morphology of the obtained samples and high encapsulation efficiency of core/shell/shell structured nanohybrids can be synthesized by this simple route.

**Table 2:** Effects of pyrolysis temperature on products.

Temperature (°C)	Sample	Yield of product	TEM studies	Efficiency of encapsulation
400	C-400	6.0	Fe/Fe_5_C_2_/CNTBs	96%
450	C-450	28.8	Fe/Fe_3_C/HCNTs	95%
600	C-600	24.6	Fe/Fe_3_C/CCNSs	99%

**Figure 6:**
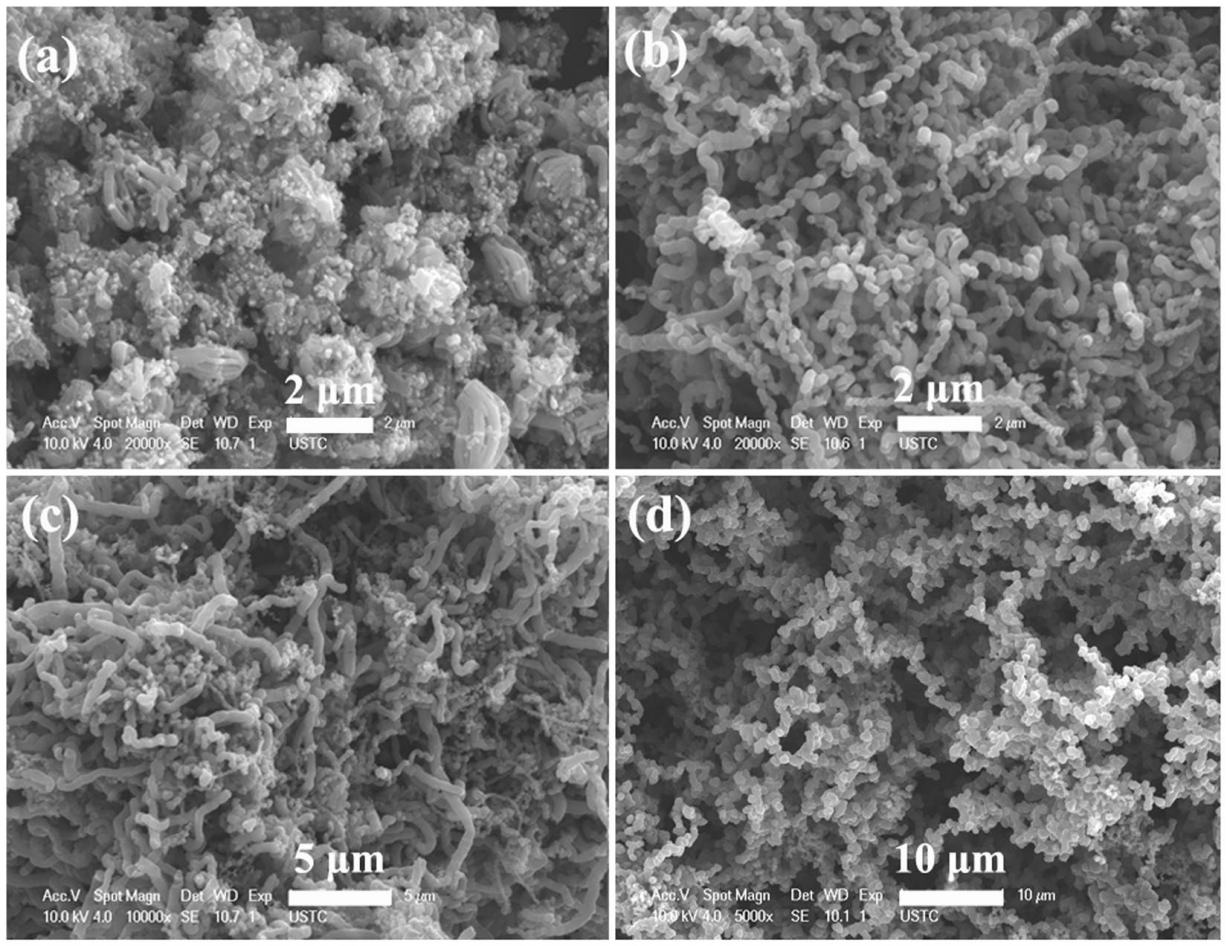
FE-SEM images of the as-prepared samples: (**a**–**c**) at 400, 450 and 600 °C without the addition of water vapor, and (**d**) at 550 °C with the water-assisted catalytic process.

In order to investigate the effect of water vapor, with other experimental conditions kept unchanged, a flow of C_2_H_2_ was introduced directly (without the supply of H_2_O) into the reaction tube and the pyrolysis of acetylene was conducted at 400, 450 and 600 °C for 2 h under atmospheric pressure, respectively. After cooling to RT, compared to those of C-400, C-450 and C-600, the yield of products obtained at 400 and 450 °C decreases greatly, while the yield of products obtained at 600 °C becomes higher. As shown in Fig. 6a–c, compared to the microstructures of C-400, C-450 and C-600, the TEM and FE-SEM investigations reveal that the introduction of H_2_O vapor has a great impact on the morphology of the sample obtained at 600 °C, and this influence on the samples obtained at 400 and 450 °C is little. In order to confirm this effect, additional comparison experiments were designed and conducted: (i) the water-assisted catalytic decomposition of acetylene at 500 and 550 °C for 2 h; (ii) the pyrolysis of acetylene at 500 and 550 °C for 2 h without the supply of H_2_O vapor. Compared with the obtained results of experiments (i) and (ii), we can find that the presence of H_2_O vapor greatly enhances the yield of samples obtained at 500 and 550 °C, and the morphological change of the sample obtained at 500 °C is unconspicuous. However, similar to that of C-600, the sample synthesized at 550 °C changes from a mixture of CNTs and HCNTs (not shown here) to high selectivity CCNSs (as shown in Fig. 6d) when the water vapor is introduced. As the results reported before^58, 59^, the introduction of water vapor can greatly improve the growth rate of carbon, which enhances the yield of the as-prepared carbon-based nanohybrids effectively. However, the effect of water vapor on the morphology of the as-prepared carbon materials is rarely reported. Generally, based on the aforementioned results, it can be seen clearly that adding a certain amount of water vapor can greatly improve the yield of the sample obtained at a relatively lower temperature (400–550 °C), and lead to the formation of Fe/Fe_3_C/CCNSs in high selectivity at a relatively higher temperature ( > 550 °C). According to the obtained results reported by Li and Liu *et al*.^60, 61^, the effect of H_2_O on the synthesis of carbon nanomaterials (CNMs) mainly exhibit two aspects: one is the water vapor can affect the surface energy of facets with higher density of atoms and increase the content of these facets effectively, which is very crucial for the CNM structure. The other is water can act as a weak oxidant for etching excessive amorphous carbon, which favors the growth of CNMs in large scale and high quality. However, excessive addition of water will severely block the formation of CNMs. Based on these reported results, the effect of water vapor on the CNM growth in this study is easily understood. However, because of the complexity, the quantitative study about the effect of water on CNM synthesis is still rarely and requires further investigation.

**Figure 7:**
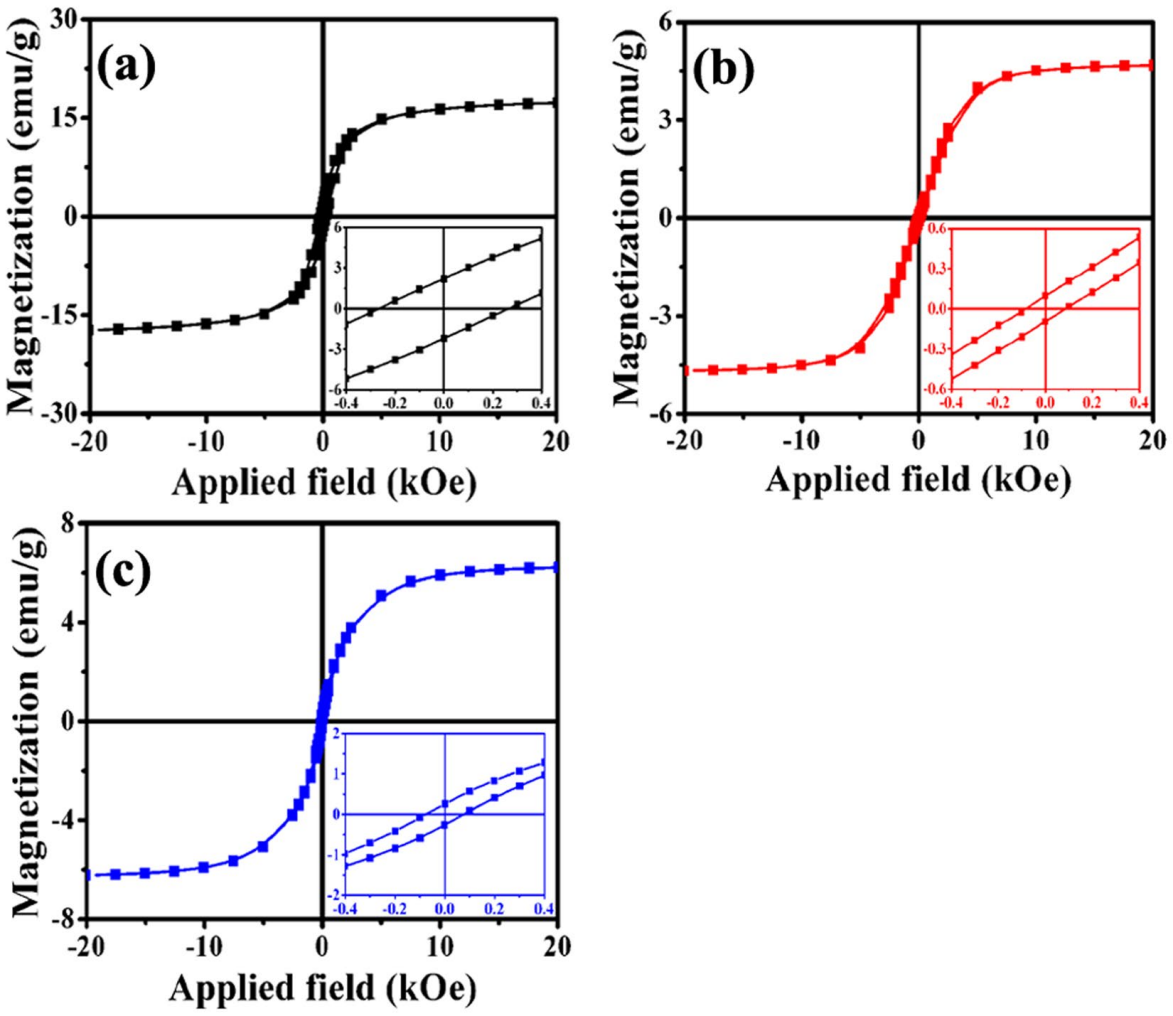
Magnetic hysteresis loop for (**a**) C-400, (**b**) C-450, and (**c**) C-600 at RT (inset is the enlarged part close to the origin).

Figure 7 displays the M-H curves of the obtained samples acquired at 300 K. As shown in Fig. 7a, the saturation magnetization (M_s_) and coercivity (H_c_) value of C-400 is 17.2 emu/g and 266 Oe, respectively. Compared to those of core/shell nanostructured carbon hybrid reported elsewhere^62, 63^, the as-synthesized Fe/Fe_5_C_2_/CNTBs shows an enhanced magnetic property due to the high content of magnetic nanoparticles. The M-H curve of the as-prepared C-450 (as shown in Fig. 7b) indicates that the M_s_ and H_c_ value of the sample is 4.7 emu/g and 84 Oe, respectively. While, as shown in Fig. 7c, the M_s_ value of C-600 is ca. 6.2 emu/g, and its H_c_ value is 74 Oe. Compared to that of C-400, the small M_s_ values of C-450 and C-600 can be ascribed to their low content of magnetic nanoparticles as mentioned before. Because of α-Fe tightly wrapped by Fe_5_C_2_/Fe_3_C and the graphitic layer, all the obtained samples showed no changes in XRD patterns and magnetic properties after being kept in air for six months, which implying that the high stabilities of the as-prepared core/shell/shell structured nanohybrids. And the good stabilities and magnetism-tunable properties of the core/shell/shell structured nanohybrids may expand their potential applications in magnetic date storage and human tumor therapy effectively.

**Figure 8:**
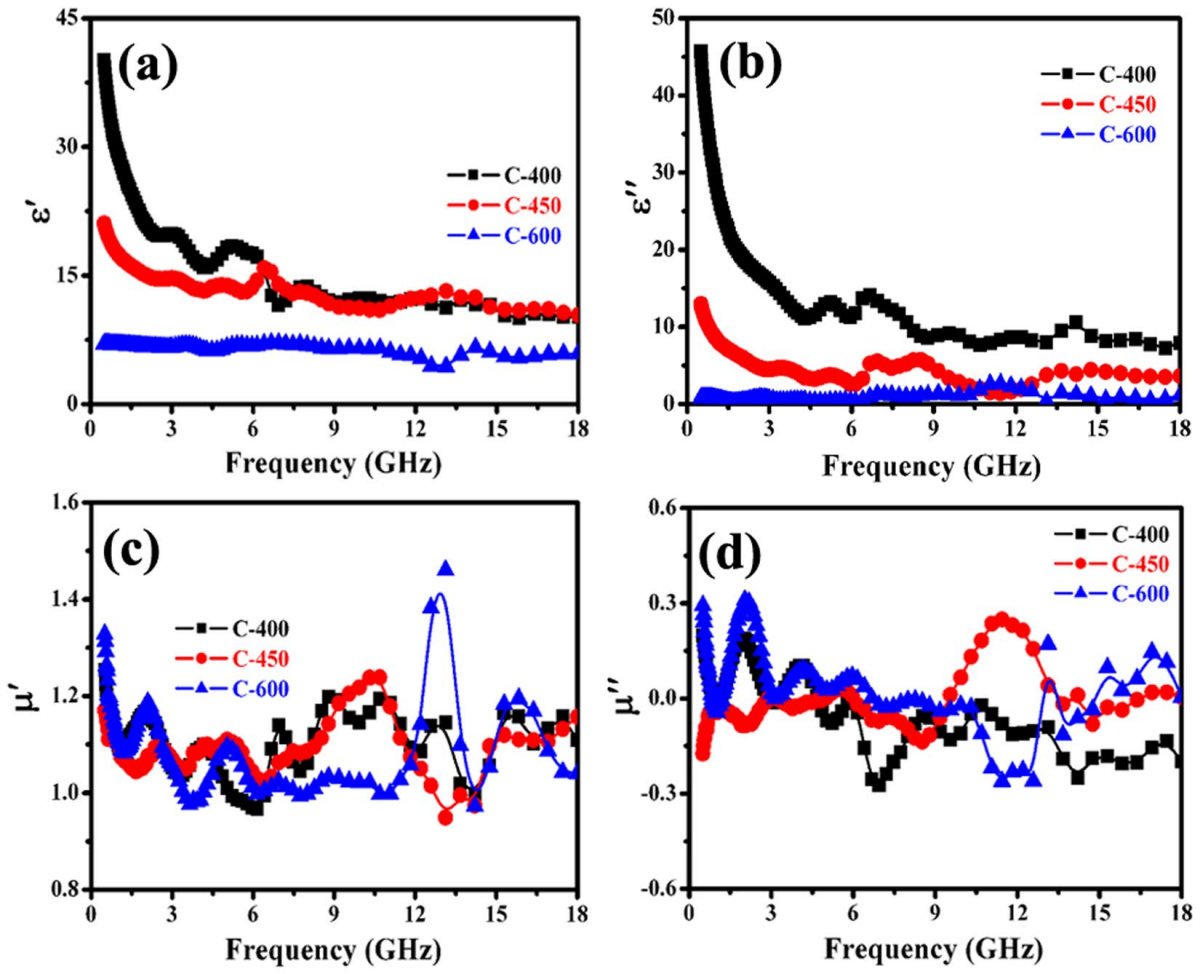
EM parameters of the obtained samples: (**a**) real part, (**b**) imaginary part of permittivity, and (**c**) real part, (**d**) imaginary part of permeability.

Figure 8 gives the complex permittivity and complex permeability of core/shell/shell structured nanohybrids in the 0.5–18.0 GHz frequency range. As shown in Fig. 8a and b, besides some fluctuations, the $\varepsilon ^{\prime}$ and $\varepsilon ^{\prime\prime}$ values of the as-prepared nanohybrids are found to decrease with the frequency in the tested region. On the basis of the Debye theory, $\varepsilon ^{\prime}$ and $\varepsilon ^{\prime\prime}$ can be described as^64^:

1 $${\varepsilon }^{\prime} ={\varepsilon }_{\infty }+\frac{{\varepsilon }_{s}-{\varepsilon }_{\infty }}{1+{\omega }^{2}{\tau }^{2}}$$

2 $$\varepsilon ^{\prime\prime} =\frac{{\varepsilon }_{s}-{\varepsilon }_{\infty }}{1+{\omega }^{2}{\tau }^{2}}\omega \tau +\frac{{\sigma }_{ac}}{\omega {\varepsilon }_{0}}$$

Where ${\varepsilon }_{s}$ is the static permittivity, ${\varepsilon }_{\infty }$ is the relative dielectric permittivity at the high frequency limit, $\omega$ is angular frequency, $\tau$ is polarization relaxation time, ${\sigma }_{ac}$ is the alternative conductivity and ${\varepsilon }_{0}$ is the dielectric constant in vacuum. According to the equations (1) and (2), one can find that the decreases of $\varepsilon ^{\prime}$ and $\varepsilon ^{\prime\prime}$ are mainly attributed to the increase of $\omega$. Moreover, it can be seen that the complex permittivity of the obtained hybrids are as follows: C-600 < C-450 < C-400, which implies that the values of $\varepsilon ^{\prime}$ and $\varepsilon ^{\prime\prime}$ can be regulated by the pyrolysis temperature and this discrepancy can be explained that the different electric polarization and conductivity properties of the as-synthesized carbon materials^65, 66^. Figure 8c and d present the complex permeability of as-prepared nanohybrids as a function of frequency. Like the previously reported core/shell structured nanohybrids^67–69^, we can see that the values of $\mu ^{\prime}$ and $\mu ^{\prime\prime}$ have some marked fluctuations and their discrepancy is unobvious in the whole frequency range, which may be related to the uneven size of catalyst nanoparticles and the little difference in magnetization as mentioned above. Moreover, we can notice that the $\mu ^{\prime\prime}$ values are negative in the specific frequency range, which suggests that the EM wave absorption mechanism is attributed to both magnetic loss and dielectric loss^69, 70^.

**Figure 9:**
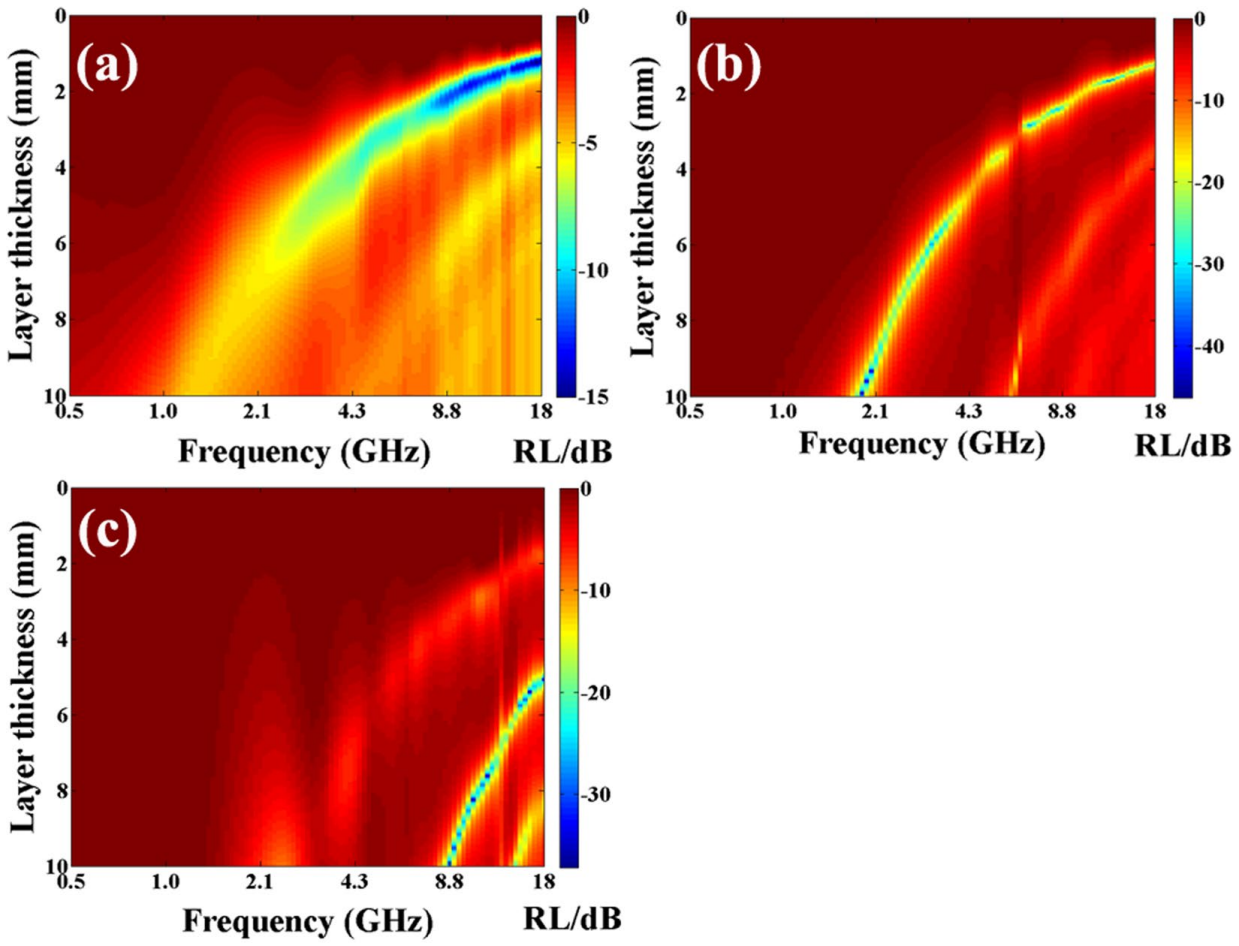
Two-dimensional representation RL values for (**a**) C-400, (**b**) C-450, and (**c**) C-600, respectively.

According to the transmission line theory, the values of reflection loss (RL) and attenuation constant ($\alpha$) are calculated by the following equations^71, 72^:

3 $${Z}_{in}=\sqrt{\frac{{\mu }_{r}}{{\varepsilon }_{r}}}\,\tanh (j\frac{2\pi fd\sqrt{{\mu }_{r}{\varepsilon }_{r}}}{c})$$

4 $$RL=20\,\mathrm{log}|\frac{{Z}_{in}-1}{{Z}_{in}+1}|$$

5 $$\alpha =\frac{\sqrt{2}\pi f}{c}\sqrt{(\mu ^{\prime\prime} \varepsilon ^{\prime\prime} -\mu ^{\prime} \varepsilon ^{\prime} )+\sqrt{{(\mu ^{\prime\prime} \varepsilon ^{\prime\prime} -\mu ^{\prime} \varepsilon ^{\prime} )}^{2}+{(\varepsilon ^{\prime} \mu ^{\prime\prime} +\varepsilon ^{\prime\prime} \mu ^{\prime} )}^{2}}}$$

where $f$ is the frequency of EM wave, *d* is the thickness of absorber, *c* is the velocity of light and ${Z}_{in}$ is the input impedance of absorber. Based on the equations (3) and (4), the RL of the as-prepard nanohybrids could be obtained and shown in Fig. 9. As shown in Fig. 9a, one can observe that the minimal RL value of C-400 is ca. −15.0 dB at 17.5 GHz with a matching thickness of 1.23 mm. And the RL values below −10 dB, which indicates 90% of EM wave energy is attenuated by the absorber, can be obtained in the frequency range of 7.7–18.0 GHz. Figure 9b shows that the optimal RL value of C-450 is ca. −46.3 dB at 1.9 GHz, and the absorption bandwidth with RL values less than −20 dB (99% of EM wave energy absorption) can be obtained in the frequency range of 1.8–3.9 and 6.7–18.0 GHz. As shown in Fig. 9c, it can be seen that the minimal RL value of C-600 is ca. −37.1 dB at 16.4 GHz, and the absorption bandwidth with RL values less than −20 dB is 9.2 GHz (from 8.8 to 18.0 GHz). Generally, as shown in Table 3, the microwave absorption performance of the obtained hybrids is as follows: C-450 > C-600 > C-400. In addition, compared to those of the representative magnetic carbon-based nanohybrids^73–80^, the as-prepared core/shell/shell structured nanohybrids here exhibit enhanced microwave absorption properties. In other words, these obtained nanohybrids can be used as the lightweight and excellent MAMs alternative to these previously reported EM wave absorption materials.

**Table 3:** Summary of the representative core/shell structured carbon-based MAMs reported in recent papers.

Sample	Optimal RL value (dB)	Frequency range (GHz) (RL < −10 dB)	Frequency range (GHz) (RL < −20 dB)	Reference
Fe/MWCNTs^a^	−39	1.0–6.0	2.04–3.47	73
Fe_3_O_4_/C	−27.9	5.0–18.0	4.5–6.5	74
Fe_3_O_4_/TiO_2_	−20.6	2.5–18.0	5.0–9.0	75
Fe_3_O_4_/CNTs	−41.6	3.0–11.4	4.4–7.5	76
Fe/HCNTs	−43.4	2.0–18.0	6.67–9.17, 15.83–18.0	77
Fe/G	−45	4.2–18.0	5.0–17.8	78
Fe/MnO_2_-G	−17.5	5.0–18.0	—	79
ZnO/Fe/Fe_3_O_4_/G	−38.4	5.0–18.0	5.9–15.2	80
Fe/Fe_5_C_2_/CNTBs (C-400)	−15.0	7.7–18.0	—	This work
Fe/Fe_3_C/HCNTs (C-450)	−46.3	1.7–18.0	1.8–3.96.7–18.0	This work
Fe/Fe_3_C/CCNSs (C-600)	−37.1	8.3–18.0	8.8–18.0	This work

Fe/multiwalled CNTs^a^.

## Discussion

**Figure 10:**
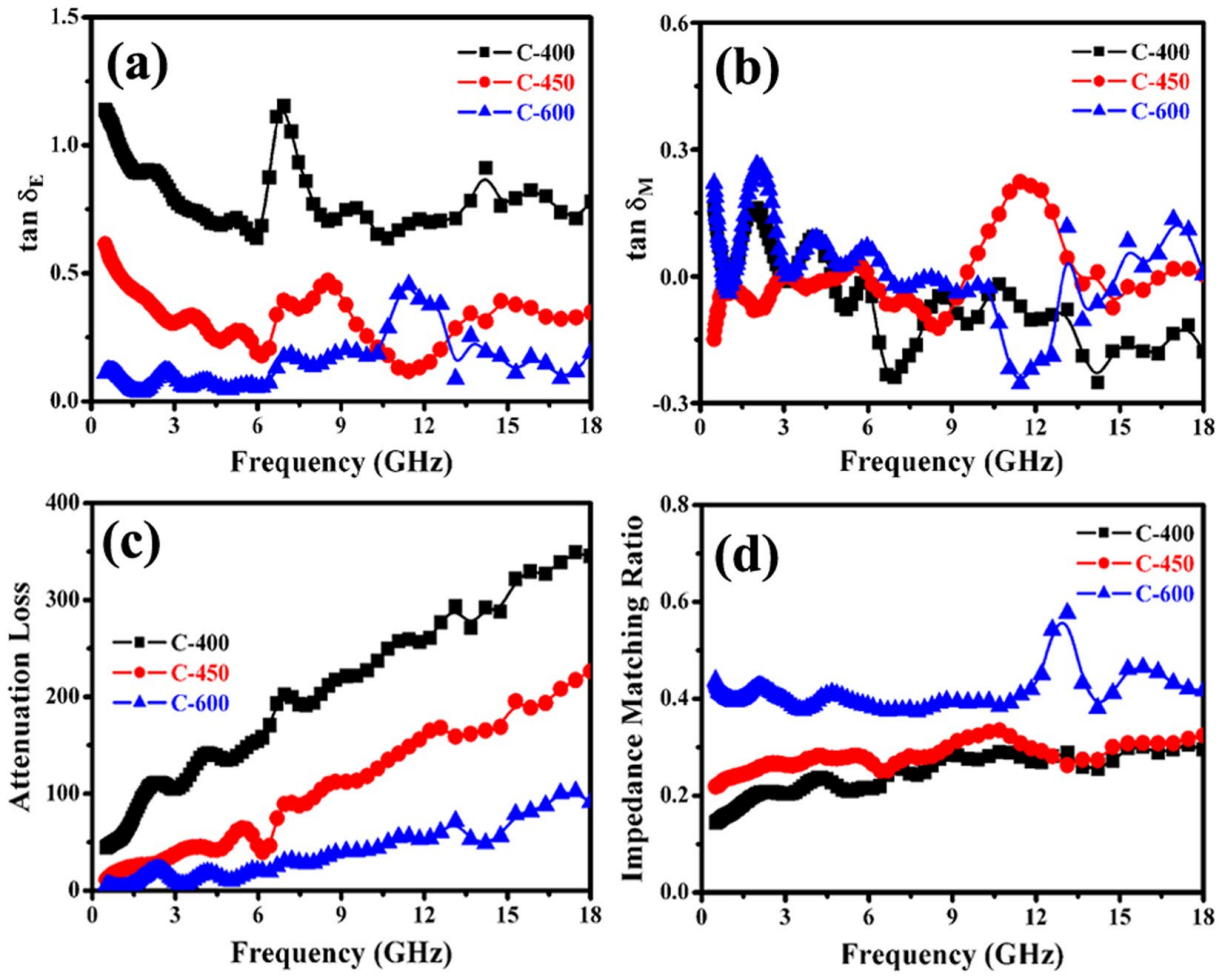
(**a**,**b**) Loss tangent, (**c**) attenuation loss, and (**d**) impedance matching of Fe/carbon-based nanohybrids.

In order to analyze the possible enhanced microwave mechanism of the as-prepared C-450 and C-600, the dielectric and magnetic loss properties, attenuation constant and impedance matching of the obtained nanohybrids were investigated in details. As shown in Fig. 10a and b, the as-prepared nanohybrids exhibit very strong dielectric loss and relatively weak magnetic loss over the whole tested frequency range, implying that the excellent EM wave attenuation is mainly due to dielectric loss. Overall, the dielectric loss performance (as shown in Fig. 10a) of the hybrids presents the following tendency: C-400 > C-450 > C-600. According to equation (5), the $\alpha$ values of the as-prepared nanohybrids are shown in Fig. 10c. It can be seen that all the as-prepared C-400 exhibits the highest $\alpha$ value while the $\alpha$ value of C-600 is the lowest. In addition, compared to the previously reported Fe/MWCNTs, Co/MWCNTs, Ni/MWCNTs and MnO_2_/Fe-G^73, 79^, the $\alpha$ values of the obtained core/shell/shell structured nanohybrids are much higher, which is conducive to improve EM wave absorption capability^72^. Based on the measured complex permittivity and permeability, the impedance matching ratios of nanohybrids are obtained and the results are displayed in Fig. 10d. Due to the contribution of magnetic nanoparticles, the impedance matching ratio of the nanohybrids presents the following tendency: C-600 > C-450 > C-400. Based on the aforementioned results, we can find that the enhanced microwave absorption capabilities of Fe/Fe_3_C/HCNTs and Fe/Fe_3_C/CCNSs are mainly attributed to the better impedance matching properties. Combined the previously reported papers with our obtained results^81, 82^, the improved impedance matching ratio of C-600 and C-450 should be related to their special structure and synergetic effect.

As shown in Fig. 9 and Table 3, one can find that the as-prepared core/shell/shell structured nanohybrids exhibit excellent microwave absorption properties. Recently, two models such as zero reflection and geometrical effect have been proposed to explain the excellent EM wave absorption properties of MAMs^9, 72^. For zero reflection, the relationship ${\mu }_{r}={\varepsilon }_{r}$ should be satisfied in terms of EM wave theory^9^. However, as shown in Fig. 8, the permittivity of the as-prepared nanohybrids is much higher than their permeability, which indicating that the obtained results cannot be interpreted by this model. As for the geometrical effect, it takes place when the incident and reflected waves in the absorbers are out of phase by 180°. This effect is strongly dependent on the quarter-wavelength equation^83^:

6 $${d}_{m}=nc/4{f}_{m}\sqrt{|{\mu }_{r}||{\varepsilon }_{r}|}\quad \quad (n=1,3,5\cdots )$$

**Figure 11:**
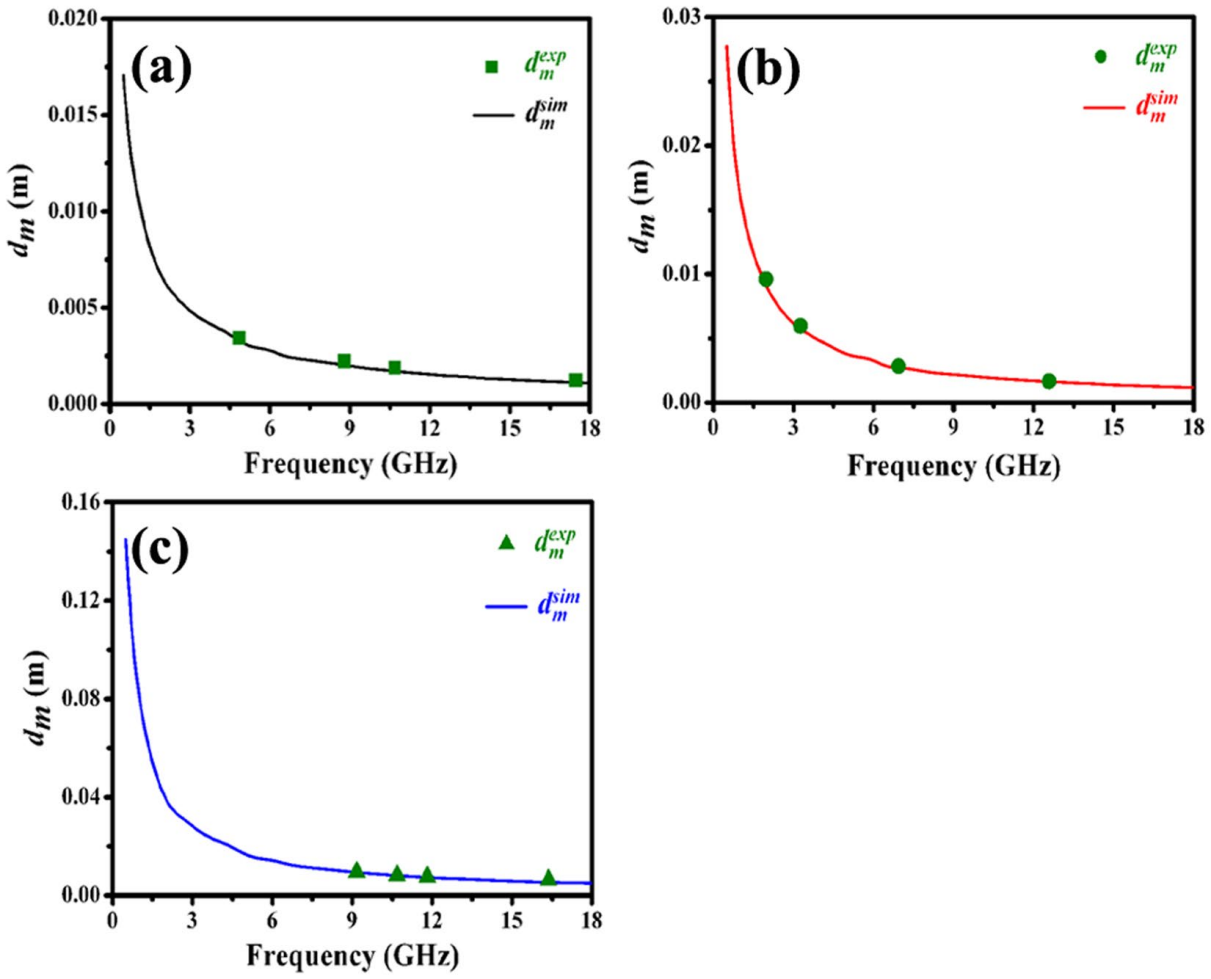
Comparison of the simulated matching thickness (${d}_{m}^{sim}$) with the ${d}_{m}^{\exp }$ obtained from RL values shown in Fig. 9.

Here, $|{\mu }_{r}|$ and $|{\varepsilon }_{r}|$ are the modulus of the measured ${\mu }_{r}$ and ${\varepsilon }_{r}$ at ${f}_{m}$, respectively. According to the model, if the matching thickness of the absorber satisfies the equation (6), the two emerging reflected EM waves from the air-absorber interface and absorber-metal interface are out of phase by 180°. And the absorber can exhibit excellent EM wave absorption property due to the extinction of EM wave on the air-absorber interface. According to equation (6), the ${d}_{m}$ can be simulated, which denoted as ${d}_{m}^{sim}$, and the results are shown in Fig. 11. It is clearly found that the obtained ${d}_{m}^{sim}$ are in good agreement with the values of ${d}_{m}^{\exp }$ (directly achieved from the RL curves in Fig. 9). Therefore, same to Fe_3_O_4_-Fe/G, Fe_3_O_4_/C and G/PANI/porous TiO_2_ reported recently^30, 84, 85^, the excellent microwave absorption properties of as-prepared nanohybrids can be explained by the quarter-wavelength matching model.

In summary, a simple and effective water-assisted approach is developed for the synthesis of core/shell/shell structured carbon-based nanohybrids with high encapsulation efficiency. By controlling the pyrolysis temperature, Fe/Fe_5_C_2_/CNTBs, Fe/Fe_3_C/HCNTs and Fe/Fe_3_C/CCNSs can be selectively synthesized in large-scale. Water vapor is considered to play an important role in the growth process. Because of α-Fe nanoparticles tightly wrapped in two layers, the obtained core/shell/shell structured nanohybrids show high stabilities and good magnetic properties. Moreover, the obtained results indicate that the as-prepared core/shell/shell structured nanohybrids exhibit excellent microwave absorption properties due to the quarter-wavelength matching model. Due to their special structure and synergetic effect, the as-prepared Fe/Fe_3_C/HCNTs and Fe/Fe_3_C/CCNSs exhibit better impedance matching and enhanced microwave absorption performances as compared to Fe/Fe_5_C_2_/CNTBs, which may be promising candidate for light-weight and high performance MAMs.

## Method

### Synthesis of catalyst precursor

Similar to the method reported previously^48^, 0.01 mol FeCl_2_·4H_2_O and 0.015 mol citric acid monohydrate were well mixed with 100 mL of absolute ethanol and stirred at 60 °C for 6 h. The mixture was heated at 80 °C until the formation of xerogel, and the obtained xerogel was then heated in air at 450 °C for 4 h for the generation of ferric oxide.

### Synthesis of core/shell/shell structured nanohybrids

As shown in Fig. 1, 50 mg of the ferric oxide powder was dispersed on a ceramic plate and placed inside a quartz reaction tube. At the beginning of the reaction, Ar gas was flowed into the quartz CVD reactor to purge the reactor before the furnace was heated up to the temperature of 450 °C. After the furnace temperature was stable, Ar was turned-off and H_2_ was allowed to the reaction chamber. After the reduction of ferric oxide in H_2_ at 450 °C for 1 h, the gas supply was shifted from H_2_ to acetylene and a flow of C_2_H_2_ through a water bubbler (heated at 40 °C in water bath) was introduced into the reaction tube and the pyrolysis of acetylene was conducted at 400, 450 and 600 °C for 2 h under atmospheric pressure, respectively. After cooling to room temperature (RT) in Ar, averagely 0.21, 1.01 and 0.86 g of the black sample could be collected in each run. For easy description, the samples generated at 400, 450 and 600 °C are denoted hereinafter as C-400, C-450 and C-600, respectively.

### Characterization

The samples were examined on an X-ray powder diffractometer (XRD) at RT for phase identification using CuK_α_ radiation (model D/Max-RA, Rigaku). The morphology investigations were examined using a field emission scanning electron microscope (FE-SEM) (model FEI Sirion 200, operated at accelerating voltages of 5 kV) and transmission electron microscope (TEM) (model Tecnai-G20, operated at an accelerating voltage of 20 kV). The X-ray photoelectron spectroscopy (XPS) data were taken on a VG Multilab2000 spectrometer. The magnetic properties of samples were measured at 300 K using a Quantum Design MPMS SQUID magnetometer (Quantum Design MPMS-XL) equipped with a superconducting magnet capable of producing fields of up to 50 kOe. For microwave measurement, 30 wt% of the as-prepared samples were mixed with paraffin and pressed into coaxial clapper in a dimension of outer diameter of 7.0 mm, inner diameter of 3.0 mm, respectively. The complex permittivity $({\varepsilon }_{r}={\varepsilon }_{r}^{^{\prime} }-j{\varepsilon }_{r}^{^{\prime\prime} })$ and complex permeability $({\mu }_{r}={\mu }_{r}^{^{\prime} }-j{\mu }_{r}^{^{\prime\prime} })$ of the composites were measured in frequency range of 0.5–18 GHz over an Agilent E8363B vector network analyzer.

## Electronic supplementary material

Supplementary file
