# Supplementary material for: Water-assisted and controllable synthesis of core/shell/shell structured carbon-based nanohybrids, and their magnetic and microwave absorption properties
Source: Sci Rep. 2017 Aug 29;7:9851. doi: 10.1038/s41598-017-10352-8 (PMC5575045; doi:10.1038/s41598-017-10352-8)
Supplement: Supplementary file 1 — Supplementary file [file 41598_2017_10352_MOESM1_ESM.doc]

**Water-assisted and controllable synthesis of core/shell/shell structured carbon-based nanohybrids, and their magnetic and microwave absorption properties**

Xiaosi Qi ^a,b,c^ Erqi Yang,^a^ Hongbo Cai,^a^ Ren Xie,^a^ Zhongchen Bai,^a,b^ Yang Jiang,^a^ Shuijie Qin,^a,b^, [[1]](#footnote-2), Wei Zhong,^c^,[[2]](#footnote-3) Youwei Du^c^

*^a^College of Physics, Guizhou University, Guiyang 550025, People’s Republic of China*

*^b^Guizhou Province Key Laboratory for Photoelectrics Technology and Application, Guizhou University, Guiyang City 550025, People’s Republic of China*

*^c^Nanjing National Laboratory of Microstructures and Jiangsu Provincial Laboratory for NanoTechnology, Nanjing University, Nanjing 210093, People’s Republic of China*


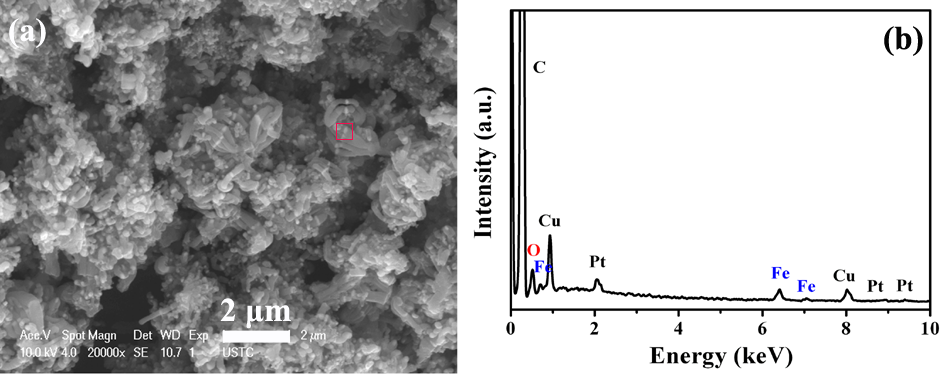


**Figure S1.** (a) FE-SEM image, and (b) EDS spectrum (the area as indicated by the red square in (a)) of C-400.


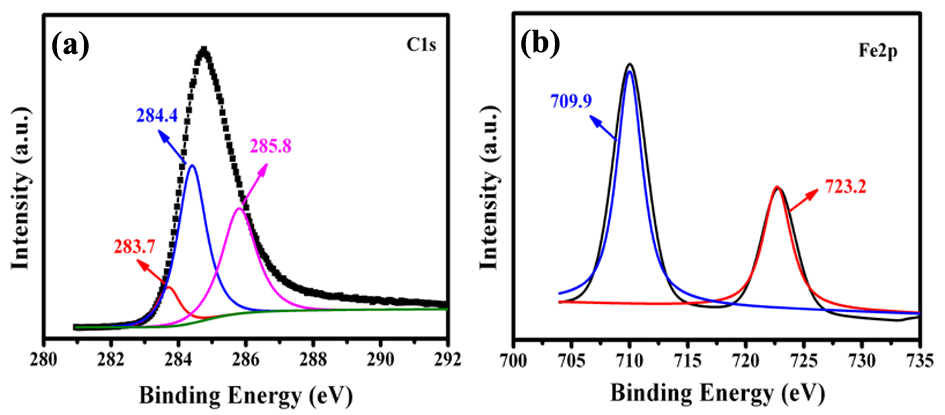


**Figure S2.** XPS spectrum of C-600 for (a) C1s, and (b) Fe2p.

1. *****Corresponding author. Phone: +86-25-83621200. Fax: +86-25-83595535

   E-mail: [wzhong@nju.edu.cn](mailto:wzhong@nju.edu.cn), shuijie_qin@sina.com [↑](#footnote-ref-2)
2. [↑](#footnote-ref-3)
